# Dor Torácica Persistente após Infecção COVID-19. Os Parâmetros Ecocardiográficos de Strain Podem Ter um Papel no Diagnóstico e na Avaliação Prognóstica?

**DOI:** 10.36660/abc.20220860

**Published:** 2023-01-24

**Authors:** Marisa Trabulo

**Affiliations:** 1 Hospital Santa Cruz Carnaxide Portugal Hospital Santa Cruz, Carnaxide – Portugal

**Keywords:** COVID-19, Ecocardiografia sob Estresse, Miocardite

Quase três anos depois do surto da pandemia de COVID-19, o número de casos graves está diminuindo com a implementação em larga escala da vacina, mas muitos efeitos colaterais a longo prazo ainda estão afetando muitos pacientes que contraíram o vírus.

Em uma metanálise de 2021,^1^os cinco efeitos de longo prazo mais comuns da infecção por COVID-19 foram fadiga (58%), dor de cabeça (44%), distúrbio de atenção (27%), perda de cabelo (25%) e dispneia (24%). A dor torácica esteve presente em 16% dos pacientes, e prevalência semelhante foi encontrada em outros estudos.^2^

Embora existam causas alternativas não cardíacas para dor torácica pós-covid, como problemas pós-respiratórios, síndrome inflamatória musculoesquelética,^3^ ou mesmo tromboembolismo pulmonar prévio, sintomas cardíacos persistentes, incluindo dor torácica, palpitações e taquicardia, podem indicar sequelas cardíacas subjacentes e merecem investigação adicional.

No artigo de Özdemir et al.^4^ mostraram uma associação entre parâmetros ecocardiográficos (SE) de strain e achados de ressonância magnética cardíaca (RMC) em pacientes com dor torácica persistente após a recuperação da infecção por COVID-19. Em comparação com a fração de ejeção do ventrículo esquerdo (FEVE), o strain global longitudinal e circunferencial teve uma relação mais forte com o envolvimento miocárdico associado ao COVID-19, especificamente miocardite prévia avaliada por RMC.

A miocardite durante a infecção ativa por covid é frequentemente subclínica e subdiagnosticada, mas sua prevalência pode ser significativa.^5^ O prognóstico a longo prazo da miocardite neste cenário ainda é desconhecido, e mais estudos são necessários para determinar se a miocardite por COVID-19 tem resultados semelhantes em comparação com a miocardite por outras causas.

Em um estudo de uma coorte de pacientes recentemente recuperados da infecção por COVID-19, a RMC revelou envolvimento cardíaco em 78 pacientes (78%) e inflamação miocárdica em curso em 60 pacientes (60%). Esta investigação não encontrou associação entre a incidência de sequelas cardíacas e a gravidade da doença aguda por coronavírus e condições pré-existentes.^6^

Em um estudo em atletas que se recuperaram de COVID-19, a RMC encontrou evidências de inflamação miocárdica em 46% dos pacientes.^7^

Embora historicamente a biópsia endomiocárdica represente o padrão-ouro diagnóstico da miocardite aguda, nos últimos anos, a RMC emergiu como a principal modalidade de diagnóstico e estratificação de risco da miocardite.

Além disso, a RMC é o padrão-ouro para quantificar os volumes biventriculares e as frações de ejeção. A RMC é recomendada em pacientes com suspeita clínica de miocardite ou dor torácica, coronárias normais e troponina elevada para o diagnóstico diferencial de origem isquêmica versus não isquêmica.^8^

O diagnóstico de RMC geralmente é baseado em uma apresentação clínica consistente com miocardite em conjunto com a presença de realce tardio pelo gadolínio (RTG) em padrões típicos e evidência de edema miocárdico na imagem T2. O RTG detectado pela RMC reflete lesão miocárdica, ou seja, necrose e fibrose.

Vários estudos de RMC mostraram o poderoso valor diagnóstico e prognóstico do RTG na miocardite.^9^

Nos últimos anos, a medição da deformação longitudinal global e regional (GLS) do ventrículo esquerdo tem sido extensivamente estudada para detectar a disfunção cardíaca em muitos distúrbios cardiovasculares. Mesmo anormalidades sutis do strain miocárdico são preditores poderosos de eventos adversos em várias doenças cardiovasculares, tendo um valor incremental para a fração de ejeção e outros fatores de risco tradicionais.^10,11^

A GLS reflete a contração longitudinal do miocárdio. Este método é mais independente do operador e mais reprodutível do que a FE.

Em pacientes com miocardite aguda e FEVE preservada, foi detectada uma redução significativa da GLS em comparação com indivíduos saudáveis. Além disso, o strain longitudinal regional acrescentou informações importantes para a localização e exstrain da lesão miocárdica. Esses achados foram favoravelmente comparados com a quantificação da RMC do realce tardio do gadolínio.^12^

Na miocardite por COVID-19, a GLS basal, a FEVE e a extensão do RTG na RMC foram preditores independentes de recuperação funcional no seguimento.^13^

A confirmação diagnóstica de miocardite, seja relacionada com a COVID-19 ou não, continuará a depender da RMC e da biópsia endomiocárdica quando indicada, mas, dadas as evidências atuais e uma vez que o acesso à RMC pode ser limitado, um método mais acessível como a avaliação de strain 2D pode desempenhar um papel na triagem e seleção de pacientes para uma avaliação posterior mais extensa.
