# Supplementary material for: Dor Torácica Persistente após Infecção COVID-19. Os Parâmetros Ecocardiográficos de Strain Podem Ter um Papel no Diagnóstico e na Avaliação Prognóstica?
Source: Arq Bras Cardiol. 2023 Jan 24;120(1):e20220860. [Article in Portuguese] doi: 10.36660/abc.20220860 (PMC10389113; doi:10.36660/abc.20220860)
Supplement: Translation

# Translation: Persistent Chest Pain after COVID-19 Infection. Can Strain Echo Parameters Play a Role in Diagnosis and Prognostication?

**Authors:** Marisa Trabulo

**Affiliations:** 1 Portugal Hospital Santa Cruz, Carnaxide – Portugal

**Keywords:** COVID-19, Stress Echocardiographies, Myocarditis

Almost three years after the outbreak of the COVID-19 pandemic, the number of serious cases is decreasing with the large-scale implementation of the vaccine, but many long-term side effects are still affecting many patients who have previously contracted the virus.

In a 2021 meta-analysis,^1^ the five most common long-term effects of COVID-19 infection were fatigue (58%), headache (44%), attention disorder (27%), hair loss (25%), and dyspnea (24%). Chest pain was present in 16% of the patients, and a similar prevalence was found in other studies.^2^

Although there are alternative non-cardiac causes for post-covid chest pain, like post-respiratory problems, musculoskeletal inflammatory syndrome,^3^ or even previous pulmonary thromboembolism, persistent cardiac symptoms, including chest pain, palpitations and tachycardia may indicate underlying cardiac sequelae and deserve further investigation.

In the article by Özdemir et al.^4^ showed an association between strain echocardiographic (SE) parameters and cardiac magnetic resonanc (CMR) findings in patients with persistent chest pain after recovery from COVID-19 infection. Compared with the left ventricular ejection fraction (LVEF), the global longitudinal and circumferential strain had a stronger relationship with long-COVID-associated myocardial involvement, specifically previous myocarditis assessed by CMR.

Myocarditis during active covid infection is frequently sub-clinical and underdiagnosed, but its prevalence may be significant.^5^ The long-term prognosis of myocarditis in this setting is still unknown, and further study is needed to determine whether COVID-19 myocarditis has similar outcomes compared with myocarditis from other causes.

In a study of a cohort of patients recently recovered from COVID-19 infection, CMR revealed cardiac involvement in 78 patients (78%) and ongoing myocardial inflammation in 60 patients (60%). This investigation found no association between the incidence of cardiac sequela and the severity of acute coronavirus illness and pre-existing conditions.^6^

An investigation of athletes who recovered from COVID-19 with CMR found evidence of myocardial inflammation in 46% of patients.^7^

While historically, endomyocardial biopsy has represented the diagnostic gold standard of acute myocarditis, over the last few years, CMR has emerged as the primary modality for diagnosis and risk stratification of myocarditis.

Furthermore, CMR is the gold standard for quantifying biventricular volumes and ejection fractions. CMR is recommended in patients with clinically suspected myocarditis or chest pain, normal coronaries, and raised troponin for the differential diagnosis of ischemic versus nonischemic origin.^8^

CMR diagnosis is often based on a clinical presentation consistent with myocarditis in conjunction with the presence of late gadolinium enhancement (LGE) in typical patterns and evidence of myocardial edema on T2 imaging. LGE detected by CMR reflects myocardial injury, i.e., necrosis and fibrosis.

Several CMR studies have shown LGE’s powerful diagnostic and prognostic value in myocarditis.^9^

In recent years, the measurement of left ventricular global and regional longitudinal strain (GLS) has been extensively studied to detect cardiac dysfunction in many cardiovascular disorders. Even subtle abnormalities of myocardial strain are powerful predictors of adverse events in numerous cardiovascular diseases, having an incremental value to ejection fraction and other traditional risk factors.^10,11^

GLS reflects the longitudinal contraction of the myocardium. This method is more operator independent and more reproducible than EF.

In patients with acute myocarditis and preserved LVEF, a significant reduction of GLS compared to healthy subjects was detected. Further, regional longitudinal strain added important information to the localization and extent of myocardial injury. These findings were favorably compared with CMR’s quantification of late gadolinium enhancement.^12^

In COVID-19 myocarditis, baseline GLS, LVEF, and the extent of LGE in CMR were independent predictors of functional recovery in the follow-up.^13^

The diagnostic confirmation of myocarditis, whether COVID-19 or non-COVID-19 related, will continue to rely on CMR and endomyocardial biopsy when indicated, but given the current evidence and since the accessibility of CMR is often limited, a more accessible method like 2D strain evaluation may play a role in screening and selecting patients for more extensive evaluation.
